# Supplementary figures and images for: An androgen receptor-based signature to predict prognosis and identification of ORC1 as a therapeutical target for prostate adenocarcinoma
Source: PeerJ. 2024 Mar 29;12:e16850. doi: 10.7717/peerj.16850 (PMC10984180; doi:10.7717/peerj.16850)

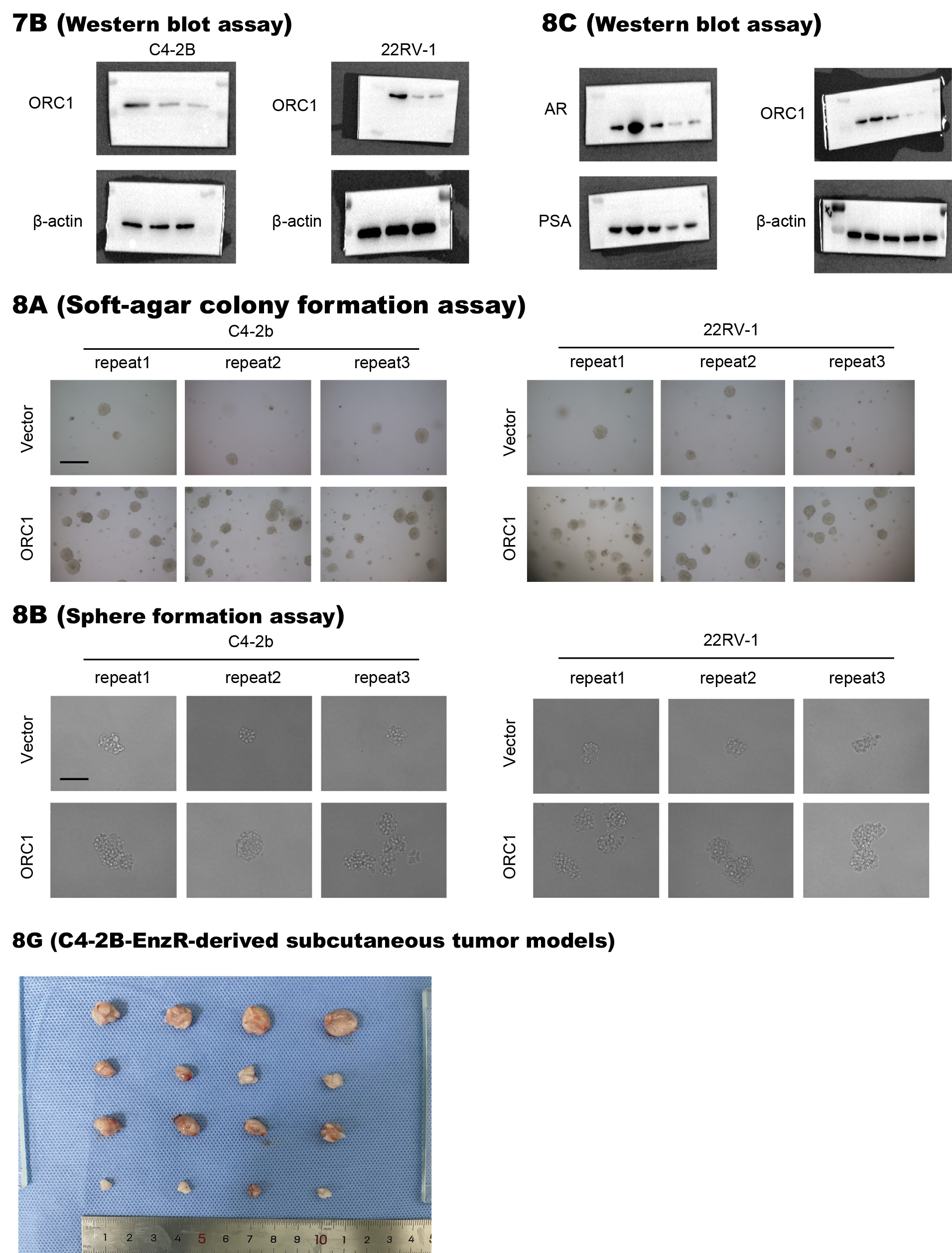

Supplement: Supplemental Information 1 [file peerj-12-16850-s001.jpg]
