# Supplementary material for: An androgen receptor-based signature to predict prognosis and identification of ORC1 as a therapeutical target for prostate adenocarcinoma
Source: PeerJ. 2024 Mar 29;12:e16850. doi: 10.7717/peerj.16850 (PMC10984180; doi:10.7717/peerj.16850)
Supplement: Supplemental Information 4 — (A) The mutational landscape reflected that mutated events occurred more frequently in high ARGs-score samples than that in low group. Besides, the Chi-square test revealed that TP53, KMT2D, FOXA1 and PTEN especially harbored more mutants compared with that in group with low risk ARGs scores. (B) Scatter plot exhibiting the associations between ARGs scores and TMB levels. (C) Kaplan-Meier analysis indicating the differential PFS survival outcomes between TMB-high and TMB-low groups. [file peerj-12-16850-s004.docx]

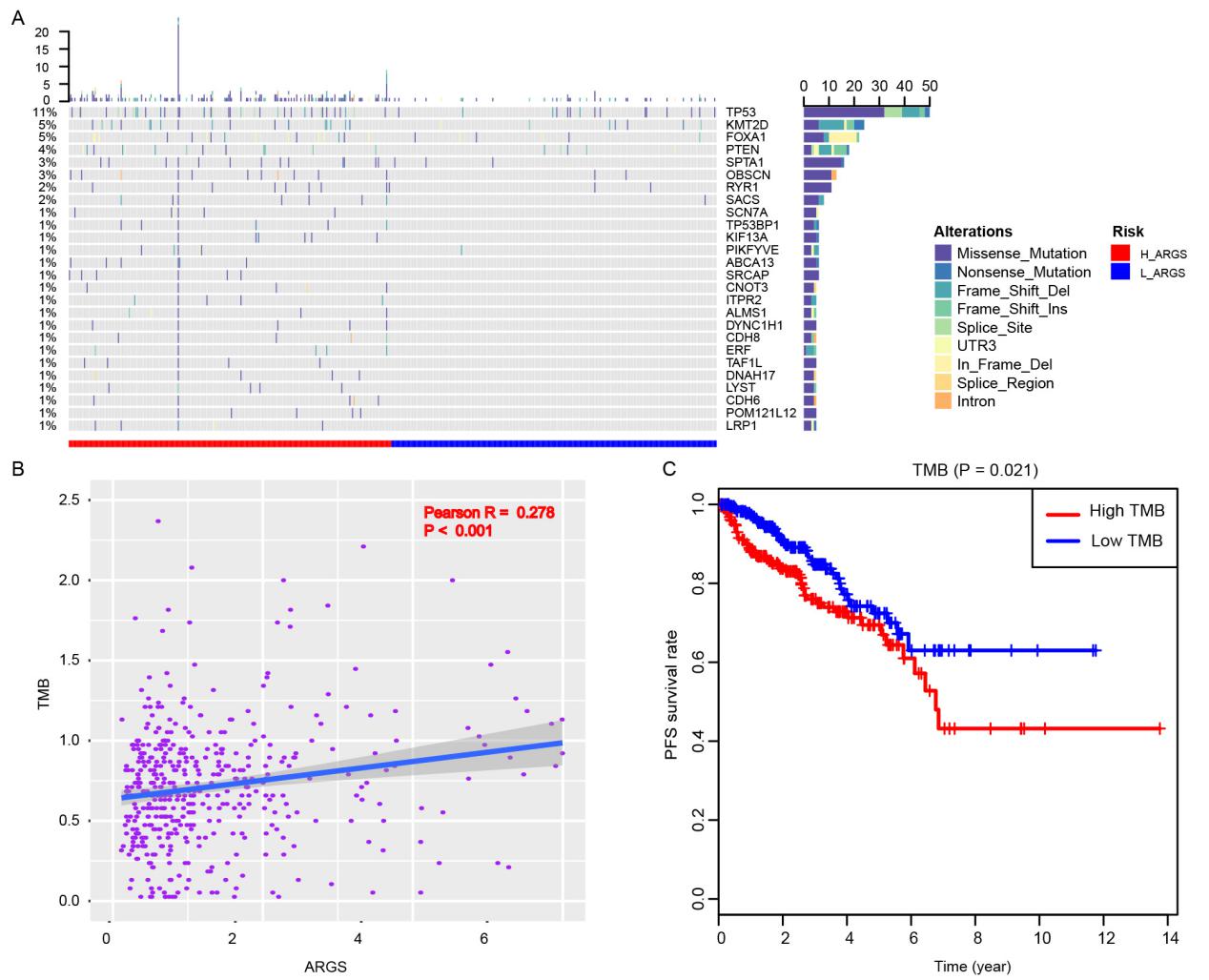


**Figure S1. Differential landscape of somatic mutation burden between high and low ARGs score levels. (A)** The mutational landscape reflected that mutated events occurred more frequently in high ARGs-score samples than that in low group. Besides, the Chi-square test revealed that TP53, KMT2D, FOXA1 and PTEN especially harbored more mutants compared with that in group with low risk ARGs scores. **(B)** Scatter plot exhibiting the associations between ARGs scores and TMB levels. **(C)** Kaplan-Meier analysis indicating the differential PFS survival outcomes between TMB-high and TMB-low groups.
